# Supplementary material for: A UMLS-based spell checker for natural language processing in vaccine safety
Source: BMC Med Inform Decis Mak. 2007 Feb 12;7:3. doi: 10.1186/1472-6947-7-3 (PMC1805499; doi:10.1186/1472-6947-7-3)
Supplement: Additional file 13 — RAP application directory. Sets up directory for RAP (RDF application for PHP) source code [file 1472-6947-7-3-S13.gz › rap/doc/tutorial/db_utils.htm]

Tutorial: RDF DB Utils


### RDF API for PHP V0.9.1

# Tutorial: RDF DB Utils

This turorial is part of the RAP - Rdf API for PHP documentation.

  

RDF DB Utils is a new graphical user interface written by Gunnar AAstrand Grimnes for dealing with DbModels.

### Contents:

|  |  |
| --- | --- |
| 1. | Configuration |
| 2. | Connecting to a Database |
| 3. | Browse through a model |
| 4. | Querying a model |
| 5. | Adding statements/models |

### 1. Configuration

The tool can be found in the tools directory under rdfdb-utils. You can specify your database settings in `config.inc.php`.
Currently we support mysql and MsAccess databases. Example: use the settings

`$i=0;   
$_DB[$i]["type"]="mysql";  
$_DB[$i]["host"]="localhost";  
$_DB[$i]["port"]="";   
$_DB[$i]["dbName"]="rdf_db";   
$_DB[$i]["username"]="root";  
$_DB[$i]["password"]="";`

for the mysql-database `rdf_db` running on `localhost`.

### 2. Connecting to a Database

You can create a new database connection or choose a connection that is specified in `config.inc.php`.

### 3. Browse through a model

You can browse through a selected model to view edit or delete statements.

### 4. Querying a model

To search in a model you can use RDQL or enter parts of the statement directly.

### 5. Adding statements/models

To add statements or models You can insert them manually or upload RDF files.
